# Water Absorption of Underwater Products by Additive Manufacturing

**DOI:** 10.3390/ma17235953

**Published:** 2024-12-05

**Authors:** Andrea Pino, Iván Ibáñez-Felip, Rosario Vidal

**Affiliations:** Institute of Advanced Materials (INAM), Universitat Jaume I (UJI), Avenida de Vicent Sos Baynat, s/n, 12071 Castelló de la Plana, Spain; apino@uji.es (A.P.); felipi@uji.es (I.I.-F.)

**Keywords:** stereolithography, water absorption, rapid prototyping, underwater applications

## Abstract

Rapid prototyping techniques offer significant advantages in terms of fabrication speed, accessibility, and low cost. This study explores the use of low-cost stereolithographic resins to produce prototypes intended for underwater conditions. The objective is to evaluate the feasibility of different low-cost resin brands by identifying their water absorption percentage and their response in terms of appearance and deformation after prolonged exposure to an underwater environment. Through three different tests, the suitability of the resins and possible coatings is evaluated, allowing for obtaining data not disclosed by commercial manufacturers and indicating that there are low-cost brands that offer water absorption levels suitable for underwater use. The coefficients for water absorption at saturation begin at 3.3% in saltwater and increase for chlorinated water. Additionally, significant insights are gained regarding the use of coatings. It is found that coatings commonly applied to filament-printed prototypes are generally less suitable for parts produced through stereolithography intended for underwater applications. The most effective strategy is to avoid using coatings altogether.

## 1. Introduction

In recent decades, additive manufacturing (AM), commonly known as 3D printing, has transitioned from a tool primarily used for rapid prototyping to an essential method for producing final parts. This shift has been driven by its growing popularity and evident advantages, such as flexibility, cost-efficiency, and the ability to create highly complex geometries [1,2]. Advances in AM have spurred the exploration of a wide range of technologies and materials, leading to its integration across numerous industries and applications. Recent years have witnessed the introduction of affordable and versatile 3D printing machines, providing designers with an increased flexibility and efficiency, particularly during the early stages of product development. AM technologies have become indispensable in engineering and innovation, especially in research environments where they are used for creating experimental prototypes [3,4,5]. Furthermore, they are being increasingly embraced by non-specialist users and hobbyists, who can now manufacture customized parts tailored to their needs using adaptable, self-optimized, and cognitive manufacturing processes [6,7].

The principal 3D printing techniques include Fused Deposition Modeling (FDM) and stereolithography (SLA). FDM, a widely accessible method, extrudes layers of thermoplastic filament to build objects, offering a cost-effective solution for functional prototypes. SLA employs a UV laser to solidify liquid resin layer by layer, enabling the production of high-resolution parts with exceptional surface finishes. These techniques have been pivotal in the rapid adoption of AM across sectors [8,9].

The applications of AM are vast, ranging from producing lightweight components in the aerospace and automotive industries to creating personalized medical devices, such as prosthetics and surgical tools. Architecture and construction benefit from its ability to create intricate models and full-scale components, while consumer industries leverage it for customized products. Despite challenges like material limitations and scalability, ongoing advancements are continually expanding the capabilities of AM, solidifying its role as a transformative tool in modern manufacturing and design [10,11].

Currently, the most accessible and user-friendly AM technologies utilize a range of materials, including thermoplastics, thermosets, metals, and ceramics [12]. Despite the rapid advancements in AM, particularly in terms of methods, materials, and the performance of manufactured parts, plastics continue to dominate due to their low cost and ease of use. However, a critical limitation persists in the technical information available from suppliers of low-cost materials, particularly regarding shrinkage and water absorption data. Moreover, much of the research conducted to date has concentrated on filament-based 3D printing [13,14], leaving a gap in understanding the behavior of resin-based materials.

In this context, there is limited research specifically addressing the development of AM products designed for high-humidity or underwater environments [15,16,17,18]. Parts intended for underwater use, especially in marine environments, must withstand prolonged exposure to aquatic, saline, and harsh climatic conditions. Therefore, the materials used must exhibit low water absorption and retain their mechanical properties under these conditions. Additionally, some applications demand the use of hydrophobic materials to ensure the internal waterproofing of the parts [19].

While several studies have explored the potential of AM for underwater applications [20], the long-term viability of materials for extended underwater exposure remains under-researched. The existing literature has primarily focused on optimizing manufacturing parameters [21] or analyzing the mechanical properties of AM materials [22]. As a result, there is a notable lack of data regarding key factors such as shrinkage and water absorption in the technical specifications of low-cost stereolithography resins. This is particularly relevant for resins intended for general use, which often lack comprehensive performance information.

This study aims to address these gaps by conducting a thorough investigation into how underwater conditions impact the impermeability, degradation, and deformation of parts manufactured using low-cost commercial resins via stereolithography. The study also assesses the viability of these resins for use in final prototypes, particularly within innovation and development contexts. By focusing on low-cost materials, this research provides valuable insights into the performance of commercially available resins under submersion, thus contributing to a broader understanding of their application potential in challenging environments.

## 2. Materials and Methods

### 2.1. Specifications of Materials, Coatings, and Solutions

For the execution of the tests, specific materials were used, which were critical to the experimental process and decisive for the results. Therefore, it is necessary to detail the properties of the resins used in the fabrication of the specimens, the coatings applied to them, and the aqueous solutions to which they were exposed.

- Resins used

This study analyzed three types of light-reactive thermosetting materials, commonly referred to as resins, intended for additive manufacturing using laser-based stereolithography (SLA). In the commercial market, resins are typically categorized based on their mechanical properties, particularly their degree of flexibility. The most commonly available resins, known as “Standard” resins, are generally more brittle and less resilient, which can present challenges in certain applications. To address these limitations, manufacturers have developed resins with improved mechanical properties, specifically tailored for engineering use. These resins, classified as “ABS-like”, offer an enhanced flexibility compared to Standard resins, making them more suitable for applications requiring a greater durability. Additionally, there are highly flexible resins, classified as “Tough. However, their flexibility can increase further when exposed to water immersion.

Based on a comprehensive evaluation of the most accessible, economical, and cost-effective options available, three resins were selected for testing. Each resin represented one of the previously defined categories and was sourced from different commercial brands and suppliers (see Table 1). The selection criteria also included compatibility with the printer used in the study. All chosen resins were priced below EUR 50 per kilogram, making them suitable for low-budget applications. This selection provides a representative sample of the broader stereolithography material market and highlights the distinct types of resins based on their properties.

- Water solutions used

For the execution of the tests, some of the samples were exposed to different types of water in order to identify how the aqueous environment affected their absorption and degradation. Four different types of water were used for this purpose, as specified in Table 2.

- Coatings specifications

For a part of this study, the specimens were treated with different types of finishes to evaluate how these affected the water absorption percentage of the stereolithography resins. Five types of finishes were used. The first corresponded to an untreated raw finish, with no coatings applied, while the remaining four finishes are specified in Table 3. These finishes were applied following the standardized procedures outlined in ISO 2812-2:2020 [24].

### 2.2. Specimen Tested

A total of 83 specimens were produced for use in three distinct experimental setups. All specimens were manufactured using the Elegoo Mars 3 3D printer from Elegoo (Shenzhen, China) and the various resins listed in Table 1.

- For the absorption experiment, 63 specimens measuring 60 × 60 × 1 mm were used, according to the dimensions and tolerances specified in ISO 294-3 [25], which specifies that the dimensions of specimens made from thermoplastic materials must meet a surface area of 60 × 60 mm and a thickness of 1.0 ± 0.1 mm. Additionally, the edges and corners adhered to the standard, being smooth and clean to prevent material loss during testing. Of these, 45 units were printed with resin R2, 9 with resin R1, and 9 with resin R3. Additionally, the absorption was also measured in specimens made from resin R2 coated with the following five different types of finishes: raw finish, polished coating, polyurethane (PU) coating, polyester resin coating (PES), and epoxy resin coating.
- For the coating experiment, 15 specimens with dimensions of 35 × 50 × 1 mm were used, all printed with resin R2. The specimens had a rectangular geometry with four holes, one at each corner, to allow for hanging during the test.
- For evaluative experiments, cylindrical specimens were tested, all of them printed with R2 resin. These hollow cylinders had a length of 26.5 mm and an internal diameter of 13.25 mm. The wall thickness varied, producing five specimens with wall thicknesses of 1 mm, 1.5 mm, 2 mm, 2.5 mm, and 3 mm, respectively. The containers were sealed hermetically using commercially available thermoplastic rubber stoppers with a snap-fit closure.

### 2.3. Experiments Design

For the design and execution of the experiments conducted in this study, the guidelines of the international standard ISO 62:2008 [26] were followed. The study was structured in two phases (Table 4). In the first phase, two different types of experiments were developed, aimed at analyzing the resins mentioned and the various types of coatings applied to them. In the second phase, referred to as the validation phase, the goal was to validate the results obtained in the initial phase.

- Absorption Test: The objective of this test ess to analyze the water absorption percentage at the point of saturation of each type of resin tested in the three different aqueous solutions [27], considering the various possible surface finishes the samples may exhibit.

**Figure 1:**
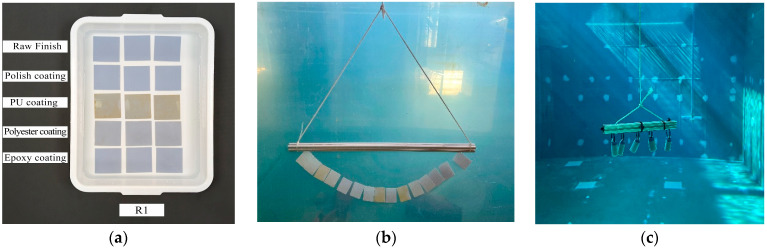
Development of the experiments: (**a**) absorption test with R2 resin, (**b**) coatings test, immersion in a 4400 L tank of 1.5 m depth, and (**c**) validation test, immersion in a 480,000 L tank of 5 m depth.

The specimens were divided into three groups based on the type of resin used in their manufacturing. For the specimens made with resin R2, they were further classified into five groups depending on the type of surface finish applied, including raw finish, polish coating, PU coating, polyester coating, and epoxy coating. For all other specimens made with resins R1 and R3, a raw finish was used. In this test, the specimens were submerged in three tanks measuring 340 × 270 × 70 mm and containing 6.5 L of distilled, chlorinated, and seawater at 23 °C (Figure 1a). These tanks had a specific geometry to avoid the contact of the specimens with the walls of the vessel. Before conducting the test, any contaminants on the surfaces of the specimens that could influence water absorption were removed using a cleaning agent that did not degrade the plastic. The degree of degradation was determined in accordance with ISO 175 [28]. After cleaning, the specimens were dried at 23 °C and a 50% relative humidity for 1–2 days. After drying in the oven, they were placed in the desiccator for another day.

For the test, the specimens were submerged in the solutions for progressively longer time intervals, following a doubling sequence (24 h, 48 h, 96 h, 192 h, etc.), as outlined by the standard. Between each interval, the samples were removed, cleaned, dried on the surface, measured, weighed, and returned to the solution. The experiment concluded when the weight of the specimens stabilized.

The environmental conditions were kept constant in a controlled environment of 23.0 ± 2.0 °C, and the solution was stirred once a day and renewed weekly.

ISO 62:2008 [26] also allows for the determination of the diffusion coefficient, D, assuming that there is correlation with Fick’s Laws and that there is a linear correlation between the logarithm of the ratio of the concentration for a given exposure time to the saturation concentration and the logarithm of the diffusion constant times and the exposure time.

- Coatings Test: This test evaluated the degradation of the coatings applied to parts manufactured using stereolithography and resin R2, with the objective of determining the best finish among the five possible options in terms of waterproofing and sealing performance.

The specimens were divided into three groups, each consisting of 5 samples. Within each group, a specimen with a different type of finish (raw finish, polish coating, PU coating, polyester coating, or epoxy coating) was included. Each group was immersed in water W4 in a 4400 L tank measuring 2 × 2 × 1.5 m.

For the test, the specimens were placed on supports, tied together with thread, and submerged, leaving a 50 mm gap between the top edge of the specimens and the water surface to prevent variations in oxygen concentration from affecting the test. A minimum distance of 30 mm was maintained between the specimens and the tank walls, as well as between the specimens themselves, in accordance with the standard (Figure 1b).

Finally, the samples were left in the solution for 24 h. After this period, the samples were removed, cleaned, and subsequently evaluated.

- Validation Test: The purpose of this test was to assess the viability of resin-manufactured parts for underwater applications requiring watertightness. The aim was to evaluate whether the deformations caused by water absorption in the material were significant enough to impact the geometry that ensured the sealing integrity of a container.

For this test, cylindrical specimens with varying wall thicknesses were placed on a support structure and submerged for 72 h in water type W4 within a 480,000 L tank, measuring 8 × 12 × 5 m, used for the operational testing of sensors and robotic systems at the Underwater Robotics and Technologies Research Center of the Universitat Jaume I (Figure 1c). After the exposure period, the samples were dried with absorbent paper and then opened to inspect for any water ingress or leakage inside the specimens.

### 2.4. Quantification

The quantification and assessment of the results were conducted using distinct methodologies tailored to each specific test. For the absorption test, a quantitative approach was employed, quantifying the amount of water absorbed by the specimen. This was determined by calculating the mass variation, defined as the difference between the initial mass and the mass measured after exposure to the aqueous solutions at the conclusion of the test and expressed as a percentage of the initial mass. For mass measurements, the specimens were heated in a forced-air convection oven, maintained at 50.0 ± 2.0 °C, to eliminate any surface and ambient moisture. Afterward, the specimens were weighed using an Ohaus analytical balance with a resolution of ±0.0001 g.

For the coatings test, the specimens were analyzed and classified according to the identified defects, following a qualitative method based on the EN ISO 4628-1:2016 standard [29]. This standard establishes seven volumes for the evaluation of the most common types of defects that may appear in a coating, using graphic reference standards. For this study, only Part 2 of this standard was applied, which assesses the degree of blistering. Microscopic analysis was performed using an Olympus SZ-PT stereoscopic zoom microscope (Olympus corporation, Tokyo, Japan), connected to a computer for image capture and subsequent evaluation. The defects found were compared with the graphical reference patterns provided in Annex A of the standard and classified using the specified code. This code categorizes defects on a five-level scale, assessing both the quantity and density of defects (represented by the first digit) and the size of these defects (represented by the second value)

For the validation test, a two-phase visual inspection method was conducted. First, the surfaces of the specimens were examined for any signs of deterioration and deformation. In the second phase, the specimens were opened by removing the stoppers to check for water ingress into the interior. The results of this test were determined using a binary response system for each phase, where “Yes” indicates the presence of deformations in the first phase and water ingress in the second phase, while “No” corresponds to the absence of deformations and no water leakage.

### 2.5. Statistical Analysis

A factorial analysis of variance (ANOVA) was performed on the results of the water absorption percentage at saturation, considering the following three independent factors simultaneously: type of water, type of finish, and type of resin. The analysis was conducted using SPSS software (IBM SPSS Statistics, Version 29.0.1.1 (244)) at a 95% confidence level, with *p* < 0.05 indicating statistical significance. The univariate procedure was employed, along with Levene’s test to assess the homogeneity of variances required for ANOVA and the Tukey post hoc test to determine the specific significant differences.

## 3. Results

**Figure 2:**
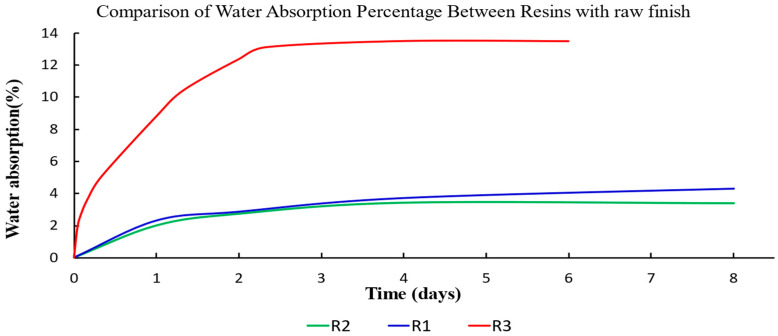
Water absorption at saturation, in percentage, for unfinished samples based on resin type in marine water.

**Figure 3:**
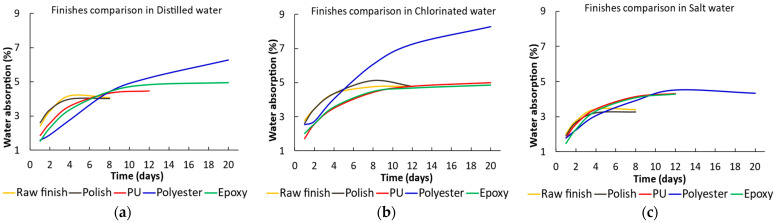
Water absorption at saturation, in percentages, of parts manufactured with R2 resin, according to the different finishes and coatings applied, in the various immersion media: (**a**) distilled water, (**b**) chlorinated water, and (**c**) seawater.

Following the sequence of the experiments, the results of the absorption test were obtained first. Initially, the water absorption percentage of the samples in their unfinished state was analyzed. This approach allowed for a comparison of the three different types of resin tested with the same finish and in the same aqueous solution (Figure 2 and Figure 3). Figure 2 shows the water absorption percentage in marine water, which was higher for R3 resin from the Conjure brand, reaching a mass variation of 12.37% in 2.5 days. After this period, its mass stabilized, reaching 13.49% over the remaining 6 days. Resins R1 and R2 exhibited lower water absorption percentages of 3.4% and 4.3%, respectively. While resin R2 stabilized within four days, resin R1 did not reach saturation until after eight days.

The following section examines how different types of finishes affected the water absorption percentage, considering the various solutions to which the specimens were exposed. This analysis was based on parts manufactured using R2 resin.

Generally, specimens with unfinished surfaces and polishing coatings stabilized their mass variation quicker, achieving a water absorption percentage between 3% and 4% (Figure 3a,c). In contrast, specimens with PU, polyester, and epoxy coatings exhibited higher absorption values and longer stabilization times, regardless of the type of medium in which they were immersed. It is noteworthy that the stabilization times were greater when samples were in a chlorinated medium, with water absorption percentages rising to between 4% and 9% (Figure 3b). Conversely, in a saline medium, the water absorption percentages tended to decrease.

**Figure 4:**
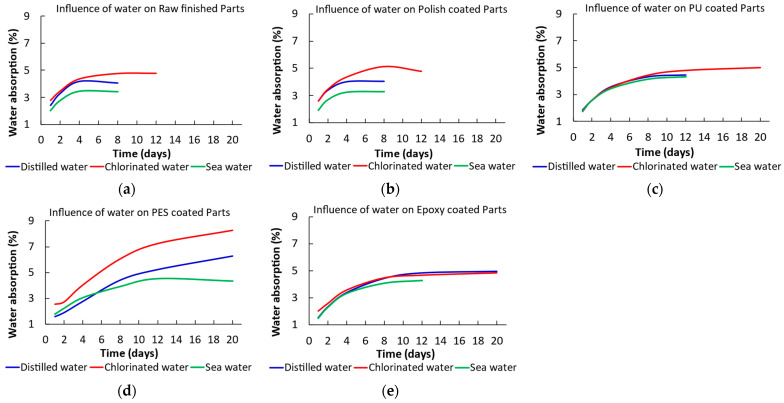
Percentage of water absorption at saturation for resin R2 according to the exposure medium for (**a**) raw finished parts, (**b**) polish-coated parts, (**c**) PU-coated partes, (**d**) polyester-coated parts, and (**e**) epoxy-coated parts.

The impact of the type of medium on each of the different finishes and coatings applied to parts manufactured with R2 resin is studied in greater detail in Figure 4.

Generally, it was observed that finishes exposed to chlorinated waters exhibited longer stabilization times and higher absorption percentages (Figure 4a–c), consistent with the results mentioned in Figure 3. In contrast, parts exposed to seawater generally showed lower values, regardless of the coating. Notably, the PU and epoxy finishes presented highly similar values across all mediums to which they were exposed (Figure 4c,e). Likewise, the polyester coating presented the highest values, regardless of the medium (Figure 4d).

Subsequently, from these data, the average absorption values were obtained for the total testing time until mass stabilization was reached, considering the different types of coatings and the various aqueous solutions used (Table 5).

The experimental data were statistically analyzed to determine whether significant differences existed between the populations. The summary of the ANOVA in Table 6 compiles the sources of variation (including the three independent factors and their interactions), degrees of freedom (dfs), F statistics, critical significance levels (Sig.) associated with each F statistic, partial eta squared, and observed power for a computed alpha of 0.05. The “Corrected Model” row encompasses all effects of the model combined. The critical level associated with its F statistic (*p* = 0.000 < 0.05) indicates that the corrected model explains a significant portion of the observed variation in the dependent variable. All sources of variation, including their interactions, also exhibit critical levels below 0.001, indicating significantly different values. Estimates regarding the extent to which each factor or combination of factors affected the dependent variable reveal high η^2^ coefficients for all sources of variation, with the lowest corresponding to the finishing factor (0.798) (Table 6). In all cases, the power is equal to 1, suggesting that the test was highly effective in detecting real differences. The coefficient of determination, or R^2^, is reported as 0.998, indicating a very high predictive capacity of the model.

Upon applying Levene’s test, a statistic of 1.32 was obtained, resulting in a right-tail probability of 0.22. Therefore, we conclude that the null hypothesis of homogeneity of variances should not be rejected, thereby validating one of the structural hypotheses of the proposed model. With the hypothesis of equal variances verified, post hoc comparisons were conducted using the Tukey test. The mean differences between the various resins and types of water concerning the dependent variable were significant (*p* < 0.001). The differences among the various types of finishes were also significant, except between the PU and epoxy finishes.

The coating test results were analyzed after obtaining the results from the water absorption tests. Initially, a visual inspection was conducted after the immersion period. Defects were observed in the specimens coated with PU, where wrinkles formed on the surface of the coating. In the specimens finished with polyester and epoxy, some blistering was noted on the surface. Finally, in the specimens with raw and polish finishes, no changes in shape or color were detected. Based on this, it was observed that the noticeable defects were very similar in shape, size, and density across all the samples of each coating.

**Figure 5:**
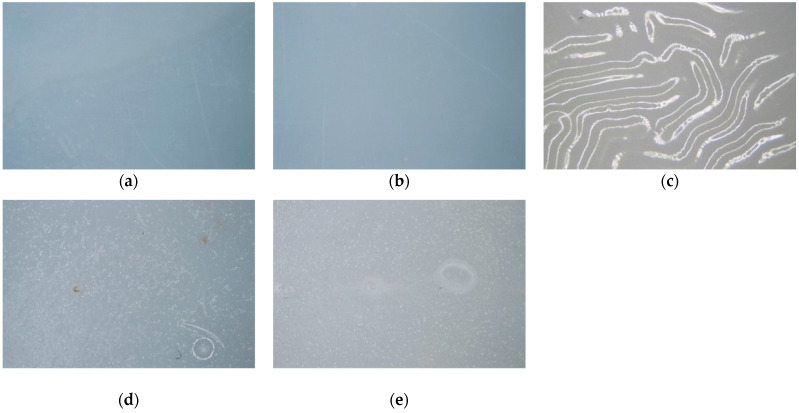
As follows, 14.8 mm diagonal: (**a**) raw, (**b**) polish, (**c**) PU, (**d**) polyester, and (**e**) epoxy.

Following the visual inspection, further results were obtained from a microscopic analysis of the samples, conducted in accordance with the standard. When analyzing the images taken at 10× magnification, no imperfections were detected microscopically in the samples with raw and polish finishes (Figure 5a,b), consistent with the previous visual inspection. However, imperfections were observed in the PU-, polyester-, and epoxy-coated samples, with a higher density of blisters. The PU-coated samples showed a lower density of imperfections, but with much larger blister sizes (Figure 5c). Finally, the polyester- and epoxy-coated samples presented a high density of defects with smaller blister sizes Figure 5d,e).

**Figure 6:**
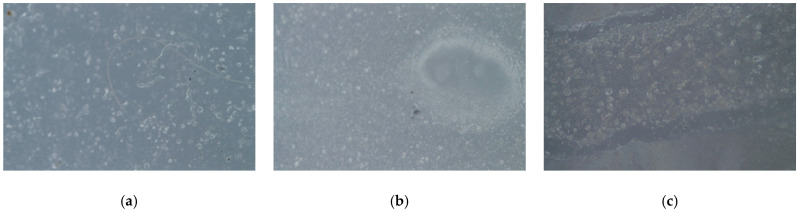
Defects magnified 3 times: (**a**) polyester specimen and (**b**) epoxy specimen, and 6 times: (**c**) epoxy specimen.

Additionally, samples were taken at a higher magnification than specified by the standard to more precisely differentiate the defects observed between the polyester resin and epoxy resin samples (Figure 6) and to rule out the appearance of other types of defects outlined in other volumes of the standard that may not have been detected in the previous phases of analysis.

The defects found were compared with the graphical reference patterns provided in Annex A of the standard and classified using the specified code (Table 7). For this analysis, a sample from each type of coating was taken as a reference, since all samples belonging to the same coating type exhibited the same type of defects.

A visual inspection of the validation test carried out with cylindrical specimens of different thicknesses immersed in chlorinated water, which had the highest water absorption coefficient, for 72 h, confirming that no water infiltration occurred in any of the cylindrical specimens. No significant deformation or appreciable changes in the appearance of the specimens were observed (Table 8).

## 4. Discussion

One of the challenges of underwater environments is the wide variety of aqueous conditions and compositions that can arise, increasing the number of potentially degrading factors for parts. This was studied using different types of aqueous solutions, simulating real-world conditions, where significant differences in water absorption percentages were observed. Generally, parts submerged in saltwater exhibited lower water absorption values compared to those submerged in distilled water and chlorinated water. Additionally, parts submerged in chlorinated water exhibited the highest absorption values, which may be related to the degrading effect of chlorine on thermoplastic and thermosetting materials, promoting the diffusion of water into the matrix [30]. Additionally, these differences were statistically significant, with significance values below 0.001 in the post hoc tests, allowing us to conclude that differences existed between the populations for the sample studied when submerged in the different types of water.

This study aimed not only to establish specific characteristics for certain low-cost resins, but also to determine which of these resins is most suitable for use in underwater applications. Among the low-cost resins readily available when conducting the study, it was found that resin R2 from the Elegoo brand showed the lowest water absorption values when used without any coatings or finishes applied. These results were also very similar to those of resin R1, from the Esun brand, identifying these two resins as the most suitable in terms of water absorption when no coating was applied. Conversely, resin R3 should be ruled out for use in underwater applications due to its high water absorption rate, which was three times higher than that of the other resins tested. This is consistent with its distinct properties, as it is the most flexible of the resins analyzed, as previously mentioned, and is supported by the statistical analysis, which concluded that there were significant differences, with a *p*-value below 0.01, indicating that there were, indeed, substantial differences in the water absorption values among the three types of resins studied.

However, in underwater applications, parts are usually finished with different types of coatings to enhance their characteristics and extend their lifespan [31,32,33]. This study analyzed how these coatings affected the water absorption percentage of the parts, and it was observed that those coated with polyester resin exhibited significantly higher absorption rates when immersed in the three different types of water. For the other four types of coatings, the results were very similar, as all absorbed a comparable percentage of water and stabilized after a similar amount of time when placed in tanks with chlorinated water or saltwater. These results were validated through statistical analysis, confirming the differences in the water absorption values among the parts treated with various coatings, with the exception of the PU and epoxy coatings, where no significant differences could be established.

So far, some types of coatings have been used inertially, due to their filling capacity, which has a very positive effect on parts manufactured using FDM that have gaps between their layers, thereby promoting the waterproofing and sealing of the parts. However, although during the first 48 h, the water absorption percentages were lower for the PU, polyester, and epoxy coatings, these took longer to stabilize, and their water absorption rates could increase significantly over longer exposure periods. It has been demonstrated that the hydrothermal behavior of polymeric composites follows a Fickian nature, which explains the increased water absorption observed in the polyester, epoxy, and PU composites [34]. However, an additional factor may be the accumulation of water in the voids formed between the coating layers or between the coating and the part [35]. This water accumulation can lead to the degradation of the coating, resulting in the formation of microscopic cracks and pores through which water may eventually penetrate the surfaces of the printed parts. Additionally, abnormally high absorption values have been observed for PES-coated parts when submerged in chlorinated water. This phenomenon can be attributed to the impact of chlorine on the kinetic degradation mechanism. As a strong oxidizing agent, chlorine can effectively break polymer chains, potentially causing cracks in the coating that facilitate an increased water absorption [36]. Therefore, this study suggests that it is preferable not to coat the parts at all, rather than using a polyester coating, in terms of water absorption, particularly when it comes to resin-based parts that will be submerged for periods exceeding 48 h.

Moreover, the coatings applied do not aim solely to reduce the percentage of water absorption, but also to preserve the physical and aesthetic properties of the parts. Additionally, it is important that the coating itself maintains an acceptable appearance after being exposed to the degrading aquatic environment and does not excessively devalue or alter the appearance of the parts. The study observed that samples with PU, polyester, or epoxy coatings exhibited a higher number of defects, as well as changes in color, worsening the appearance and finish of the parts and deteriorating the homogeneity of the coating. In contrast, those that were not coated or were treated with polish showed an appearance almost identical to the pre-immersion phase, and, therefore, did not present any defects that were classifiable according to the standard. These results align with the findings from the absorption tests [37], indicating that the finishes that performed best in the experiments were the raw finish and the polish coating.

The range of water absorption coefficients in saturation (3.3% to 5.4%) for resins R1 and R2 across different water compositions and coatings, except for polyester, is comparable (considering the high variability that can exist for the same material) to the values observed for polyamides, EVOH, acrylics, PLA, and others. More specifically, the absorption values observed in samples made with resin R2 and a raw finish fell within the range obtained for similar materials. With absorption values between 44 and 69 μg/mm^3^, these values were slightly higher than those reported for injection-molded polyamides, which have an absorption of 30 μg/mm^3^ [38,39], and lower than those observed for laser-sintered (LS) polyamides, which exhibit 10% absorption values [40].

However, these values were higher than those of other thermoplastics such as ABS, PC, PET, PE, and PP, which have water absorption rates below 1% [41]. These thermoplastics can be used as filaments for the FDM printing technique. Nevertheless, our experience in designing and prototyping underwater applications using FDM printing has been significantly less favorable compared to stereolithography with resins R1 and R2. This is primarily due to the considerable difficulty, with low-cost equipment, in achieving the perfect fusion of filaments necessary to prevent water from penetrating the designs.

Finally, a validation test was conducted to assess whether water absorption and material degradation could affect the parts in terms of deformation, potentially compromising the design quality and watertightness. Since a specimen thickness of 1 mm was used in the absorption study, this value was set as the minimum required thickness for the validation test. Absorption tests conducted over a 72 h period with resin R2 confirmed that there were no deformations compromising watertightness, nor was there any water infiltration through the material.

This study arose from the design needs that emerged during the development of prototypes in an experimental phase. As a result, the study is limited by the premise of using low-cost materials and machinery, as well as by the available stereolithography printer, which is a very low-cost model with reduced dimensions. Access was restricted to the least expensive resins available on the market at the time of the experiment’s planning. It should be noted that this market is booming, so advancements in materials and information about them are increasing quickly. Therefore, as future work, a more exhaustive study could be conducted that expands the number of analyzed resins, including those with higher prices. Another line of work could involve long-term exposure, subjecting the parts to much longer immersion periods and studying the reaction of the resins and coatings to the appearance of biofilm.

Finally, the results obtained suggest that none of the applied coatings improved the absorption properties or the appearance of parts that were manufactured with resin through stereolithography and submerged in saltwater, chlorinated water, or distilled water. This provides information for the development of subsequent designs.

## 5. Conclusions

This study provides evidence supporting the suitability of low-cost resins used in rapid prototyping via stereolithography for underwater use across various marine environments. The findings demonstrate that parts manufactured with generic resins through this technique can be effectively used for applications requiring a thickness exceeding 1 mm, while maintaining water absorption at a saturation below 5.4%. Furthermore, the study suggests that parts produced with stereolithography using generic resins do not require additional coatings, as they do not undergo deformation, visible surface changes, or defects in an unpolished state, even after 78 h of submersion.

These results have direct implications for the design of parts for underwater applications, indicating that to achieve low-cost, highly water-resistant parts with a strong durability in humid environments, generic resins with an unpolished finish are the most effective choice. The use of epoxy, polyester, or PU coatings is discouraged, as these may compromise appearance and water absorption without providing notable advantages.

## Figures and Tables

**Table 1:** Classification and relevant technical specifications of the resins used and analyzed in this study.

ID	Model	Brand	Density (g/cm^3^)	Elastic Modulus (MPa)
R1	LCD Standard Resin	Esun, Shenzhen, China	1.08–1.13	1000
R2	ABS-Like Photopolymer Resin	Elegoo, Shenzhen, China	1.05–1.25	1882
R3	JAMG HE Tough	Conjure, Shenzhen, China	1.08–1.13	310

**Table 2:** Properties of water used in absorption tests.

ID	Water Type	Free Chlorine (mg/L)	Total Chlorine (mg/L)	Salinity (PSU)	T (°C)
W1	Distilled water	-	-	-	23
W2	Chlorinated water	0.36	0.35	-	23
W3	Seawater	-	-	38.1 [23]	23
W4	Cirtesu tank water	3.76	3.95	-	26

**Table 3:** Identification of commercial coatings tested and compared in this study.

Coating Type	Material	Brand	Proveedor
Polish coating	081—Polishing Gloss Enhancer	Bruguer	AkzoNobel Coatings, S.L., Amsterdam, The Netherlands
PU coating	Marine Varnish	Hempel	Hempel Paints, S.A.U., Köge, Denmark
Polyester coating	Polyester resin	Quimibase 2000	QUIMIBASE 2000, S.L., Barcelona, Spain
Epoxy coating	Epoxy resin 605—A	Quimibase 2000	QUIMIBASE 2000, S.L., Barcelona, Spain

**Table 4:** Experimental planning detailing the various types of resins, finishes, and geometries examined in the study.

Experiment Type	Resin Type	Water Type	Specimen Coating	Specimen Geometry	Units
Absorption	R2	W1	Raw finish	Square (60 × 60 × 1 mm)	3
			Polish coating		3
			PU coating		3
			Polyester coating		3
			Epoxy coating		3
		W2	Raw finish		3
			Polish coating		3
			PU coating		3
			Polyester coating		3
			Epoxy coating		3
		W3	Raw finish		3
			Polish coating		3
			PU coating		3
			Polyester coating		3
			Epoxy coating		3
	R1	W1	Raw finish		3
		W2	Raw finish		3
		W3	Raw finish		3
	R3	W1	Raw finish		3
		W2	Raw finish		3
		W3	Raw finish		3
Coating	R2	W4	Raw finish	Rectangular (35 × 50 × 1 mm)	3
			Polish coating		3
			PU coating		3
			Polyester coating		3
			Epoxy coating		3
Validation	R2	W4	Raw finish	Cylindrical (Multiple thicknesses)	5

**Table 5:** Results of the coefficients of water absorption at saturation.

	Raw Finish						Polish Coating		PU Coating		Polyester Coating		Epoxy Coating	
	R1		R2		R3		R2		R2		R2		R2	
	Mean (%)	SD	Mean (%)	SD	Mean (%)	SD	Mean (%)	SD	Mean (%)	SD	Mean (%)	SD	Mean (%)	SD
Distilled water	5.022	0.252	4.062	0.072	17.444	0.201	4.029	0.113	4.469	0.375	6.272	0.033	4.957	0.051
Chlorinated water	5.397	0.063	4.769	0.032	13.474	0.479	4.786	0.027	4.991	0.058	8.291	0.278	4.853	0.165
Seawater	4.304	0.015	3.409	0.023	13.489	0.068	3.272	0.169	4.301	0.127	4.324	0.476	4.296	0.094

**Table 6:** Tests of between-subjects effects for the dependent variable water absorption at saturation. R^2^ = 0.998. Significance at 0.05. (*) means the interaction between both terms.

Source	df	F	Sig	Partial η^2^	Observed Power
Corrected model	20	944.969	0.000	0.998	1.000
Intercept	1	45,453.566	0.000	0.999	1.000
Finishing	4	41.547	0.000	0.798	1.000
Water type	2	150.771	0.000	0.878	1.000
Resin type	2	6818.343	0.000	0.997	1.000
Finishing * Water type	8	25.642	0.000	0.830	1.000
Water type * Resin type	4	116.136	0.000	0.917	1.000
Error	42				1.000
Total	63				1.000
Corrected Total	62				1.000

**Table 7:** Results of blistering degree of the coatings obtained by comparing graphic patterns according to the UNE 4628-2:2026 standard [29].

Specimen	Finishing Type	Blistering Degree
1	Raw finish	0 (0)
2	Polish coating	0 (0)
3	PU coating	3 (S5)
4	Polyester coating	4 (S3)
5	Epoxy coating	4 (S2)

**Table 8:** Results of the validation test with cylindrical specimens of different thicknesses after 72 h underwater.

Specimen	Thickness (mm)	Geometric Deformations	Appearance Changes
1	1	No	No
2	1.5	No	No
3	2	No	No
4	2.5	No	No
5	3	No	No

## Data Availability

The original contributions presented in this study are included in the article. Further inquiries can be directed to the corresponding author.
